# The miRNA Expression Profile of Experimental Autoimmune Encephalomyelitis Reveals Novel Potential Disease Biomarkers

**DOI:** 10.3390/ijms19123990

**Published:** 2018-12-11

**Authors:** Shivaprasad H. Venkatesha, Steven Dudics, Yang Song, Anup Mahurkar, Kamal D. Moudgil

**Affiliations:** 1 Department of Microbiology and Immunology, University of Maryland School of Medicine, Baltimore, MD 21201, USA; hvshivaprasad@gmail.com (S.H.V.); sdudics1@gmail.com (S.D.); 2 Institute of Genomic Sciences, University of Maryland School of Medicine, Baltimore, MD 21201, USA; ysong@som.umaryland.edu (Y.S.); amahurkar@som.umaryland.edu (A.M.); 3 Division of Rheumatology, Department of Medicine, University of Maryland School of Medicine, Baltimore, MD 21201, USA

**Keywords:** experimental autoimmune encephalomyelitis, inflammation, Micro-RNA, miRNA, miR-99b, miR-125a, miR-146b, miR-193b, multiple sclerosis, myelin oligodendrocyte glycoprotein (MOG), MOG_35–55_

## Abstract

Multiple sclerosis (MS) is a debilitating autoimmune disease affecting over 2.3 million people worldwide, and it is characterized by inflammation and demyelination of nerve cells. The currently available biomarkers for the diagnosis and management of MS have inherent limitations, therefore, additional new biomarkers are needed. We studied the microRNA (miRNA) profile of splenocytes of mice having experimental autoimmune encephalomyelitis (EAE), a model of human MS. A miRNA-microarray analysis revealed increased expression of nine miRNAs (let-7e, miR-23b, miR-31, miR-99b, miR-125a, miR-146b, miR-155, miR-193b, and miR-221) following EAE development. Interestingly, serum levels of miR-99b, miR-125a, and miR-146b were significantly higher in EAE mice compared to normal mice. Bioinformatics analysis revealed the experimentally validated as well as predicted gene targets of specific miRNAs that are important for disease progression in MS. Specifically, we observed inverse correlation in the levels of miR-99b versus *LIF*, and between miR-125a versus *BDNF* and *LIF*. Our results suggest that above-mentioned miRNAs may play a crucial role in the pathogenesis of MS, and that miR-99b, miR-125a, and miR-146b in particular may serve as useful biomarkers for disease activity.

## 1. Introduction

Multiple sclerosis (MS) is an autoimmune disease of the central nervous system (CNS) and it is characterized by inflammation and demyelination of neurons. MS is a multifactorial disease involving both genetic and environmental components [1]. For example, HLA-DPB1 allele and its polymorphisms are linked to MS [2,3], and environmental factors such as smoking [4] and infections [5] can influence the severity of MS. The prevalence of MS worldwide has shown a gradual increase in the past decade [6]. The highest numbers of patients afflicted with MS are in North America [7,8]. In MS, immune cells, including CD4+ T cells, CD8+ T cells, and B cells contribute to the inflammation and demyelination of the neuronal cells in the CNS [1,9,10]. Among the CD4+ T cells, IL-17-producing T helper 17 (Th17) cells play a critical role in the disease process. The CD8+ T cells can contribute via their cytotoxic activity as well as IL-17 production (Tc17 cells). The B cells can serve as antigen-presenting cells to the T cells, produce antigen-specific antibodies that can activate the Complement system, and secrete enzymes such as matrix metalloproteinase-9 that can modulate the activity of endothelial cells. Currently, there are three main tests used to aid the clinical diagnosis and management of MS [11], namely the oligoclonal bands in the cerebrospinal fluid (CSF) [12], which are attributable mostly to immunoglobulins; the white matter/Gadolinium (Gad)-enhancing lesions detected by magnetic resonance imaging (MRI) [13,14], which correspond to active lesions with inflammation; and the John Cunningham (JC) virus antibody titers [15,16], which demonstrate exposure of the patient to this virus and the likely risk of developing progressive multifocal leukoencephalopathy (PML) following immunosuppressive therapy. While these tests have consistently been used in the clinic, they have inherent limitations of specificity and sensitivity [17,18,19]. Therefore, there is a need to identify and characterize new biomarkers for both disease development and monitoring of changes disease activity following appropriate therapy. We believe that microRNAs (miRNAs) can fill this gap and reinforce the existing tests for MS.

The miRNAs, which are short, noncoding RNA sequences, have been shown to serve as master regulators of cellular processes and are directly associated with various pathological conditions [20,21]. They exert their function by binding to the 3’ untranslated region (UTR) sequences of the target messenger RNAs (mRNAs), and either initiate their degradation or inhibit their translation [22]. Furthermore, the unique expression patterns of miRNAs or the expression levels of specific miRNAs in cancer and few other diseases coupled with their functional role have revealed the potential of miRNAs as biomarker for the diagnosis and prognosis of those diseases [23,24,25]. In addition, miRNAs could serve as potential targets for therapy [21,26]. In comparison to studies on miRNAs in cancer, there is relatively much less information on miRNAs in autoimmunity. However, increasing attention is now being directed to this area of research [27,28,29,30,31,32]. To determine the role of miRNAs in disease progression of MS as well as their utility as disease biomarkers, we used the experimental autoimmune encephalomyelitis (EAE) model of human MS.

EAE is the most commonly used model to study MS [33,34]. For EAE induction, mice (e.g., C57BL/6) are immunized with myelin antigens (e.g., myelin oligodendrocyte glycoprotein (MOG) or its peptide 35–55 (MOG_35–55_)) in an adjuvant to sensitize the T cells in the peripheral lymphoid tissue (e.g., spleen and lymph nodes). These activated T cells then traffic to the CNS and induce disease [35]. As the pathogenic autoimmune process is driven by antigens such as MOG, we examined MOG_35–55_-induced miRNAs in the splenocytes of EAE mice. (For simplicity, henceforth, MOG_35–55_ is also referred to as MOG below.) We also examined circulating serum miRNAs in EAE mice. We hypothesized that certain miRNAs play a crucial role in disease progression of EAE/MS, and that these miRNAs may be harnessed for use as biomarkers of disease development. The results of our study supporting these propositions are summarized below.

## 2. Results

### 2.1. The MOG_35–55_-Induced miRNA Expression Profile of Mice with EAE

**Figure 1:**
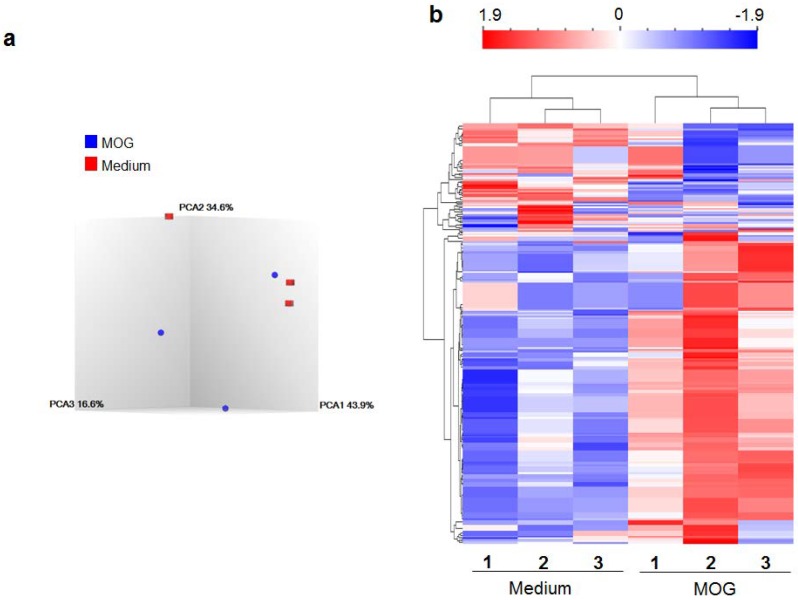
The differential expression of miRNAs in response to the disease-related antigen (MOG_35–55_). Splenocytes (SPCs) of MOG_35–55_-induced experimental autoimmune encephalomyelitis (MOG-EAE) mice were restimulated for 24 h with MOG_35–55_ (25 μg/mL) or in medium alone without MOG_35–55_ (Medium control). The total RNA isolated from these SPCs was then used for miRNA expression using GeneChip^TM^ miRNA 4.0 Array (Affymetrix, Santa Clara, CA, USA). The data was analyzed statistically as follows, (**a**) three-dimensional scatter plot of principal component analysis (PCA) displaying the relationship between the test and control groups and (**b**) heatmap and hierarchical clustering of miRNA expression profiles of MOG_35–55_-restimulated splenocytes of EAE mice and that of SPCs in medium alone (baseline). Red color indicates higher expression, whereas blue color indicates reduced expression of miRNAs. (*n* = 3 each for MOG_35–55_ restimulation and medium control).

Using the MOG_35–55_-induced EAE (MOG-EAE) model, we determined the miRNA expression profile of splenocytes (SPCs) of diseased mice. RNA was isolated from SPCs of EAE and control mice, and cultured in medium alone or medium containing MOG_35–55_ (for antigenic restimulation of the lymphoid cells). The RNA from these cells was then tested using miRNA microarray. Thereafter, following statistical analysis and setting appropriate cut-offs (*p*-value < 0.05 and fold change <−2 or > 2), we compiled the results of PCA mapping (Figure 1a) and heat map (Figure 1b) (for details of PCA, please refer to Section 4.4 under Methods). Examination of the intensity signals of control versus disease (EAE) group showed that 142 miRNA elements were significantly different between the two groups (the distinction between “miRNA elements” and “miRNA” is given under Methods section “Micro-RNA expression analysis”). A total of 129 miRNA elements were increased by antigen alone. Similarly 13 miRNA elements were decreased by antigen alone.

### 2.2. Selection of miRNAs and Their Validation by qRT-PCR and Serum miRNA Testing

**Figure 2:**
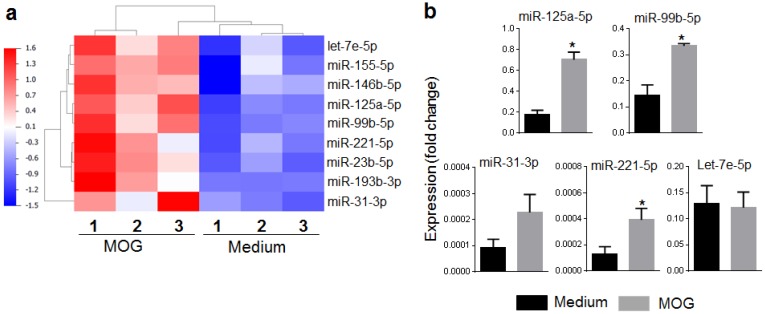
Top-listed miRNAs. (**a**) Heatmap of top-listed miRNAs showing significant difference in their expression profiles when comparing MOG_35–55_-restimulated splenocytes vs. medium control (*p* < 0.05, and fold change >2). It was derived from the heatmap shown in Figure 1. Blue color represents low, while red color represents high expression levels; (**b**) qRT-PCR validation of selected miRNAs shown in ‘a’ and presented here as ‘fold change’ over the medium control (*n* = 3–4 for different miRNAs). Total RNA was used for quantification of mature miRNAs using TaqMan MicroRNA Assays kit (Life technologies, Carlsbad, CA, USA) according to the manufacturer’s protocol. A single-stranded cDNA specific for miRNA to be tested was synthesized from total RNA, and it was amplified using sequence-specific primers. The specific miRNA levels were normalized using U6 as control and the results expressed as ‘fold change’. (* *p* < 0.05).

**Figure 3:**
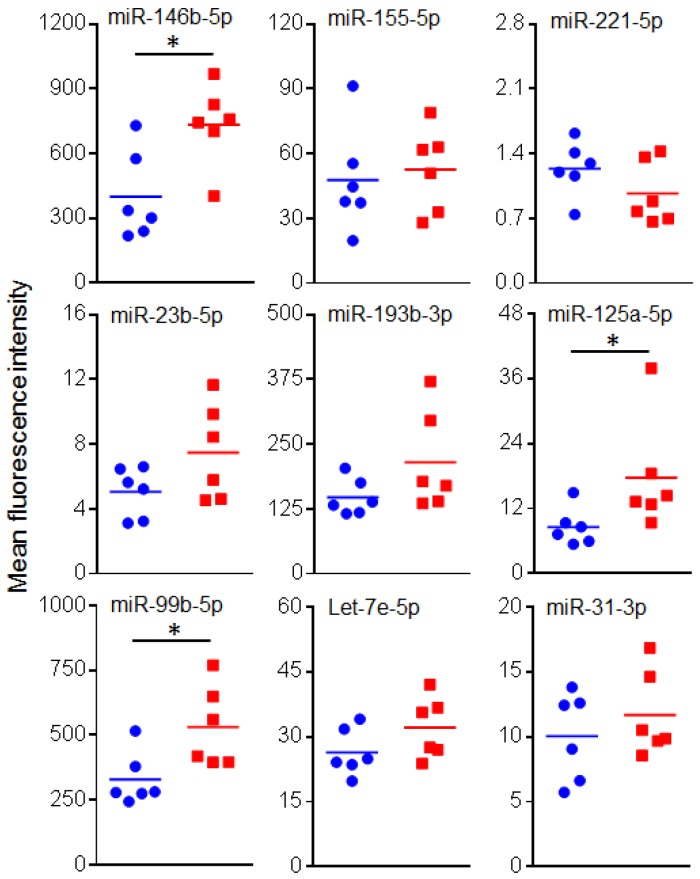
Testing miRNA levels in sera of mice. The levels of miRNAs was determined in serum samples obtained from normal (naïve) control and EAE mice (*n* = 6 each) using Multiplex miRNA assay. The data was analyzed using FirePlex Analysis Workbench software, normalized using appropriate controls and presented as mean fluorescence intensity. Blue circle: normal mice, and red square: EAE mice. (* *p* < 0.05).

After initial analysis of the data, a set of top nine mouse miRNAs (let-7e, miR-23b, miR-31, miR-99b, miR-125a, miR-146b, miR-155, miR-193b, and miR-221) was selected for further analysis (Figure 2a). A subset of these miRNAs was randomly selected for validation by quantitative Real-Time PCR (qRT-PCR) analysis of total RNA from SPCs cultured under the same conditions as those used for the microarray analysis described above. The expression of four of five miRNAs tested matched the pattern observed in microarray analysis (Figure 2b), although statistical significance was observed for three of these four miRNAs. In parallel, we tested sera of naïve and EAE mice for the same nine miRNAs identified by microarray analysis (details given below; Figure 3). Interestingly, eight of nine miRNAs (except miR-221) showed the same pattern as that observed in microarray analysis, namely an increase in EAE mice. However, the difference was statistically significant for 3 miRNAs (miR-99b, miR-125a, and miR-146b). Taken together, the results of qRT-PCR and serum testing validated the relevance of these miRNAs for further analysis.

### 2.3. Circulating miRNAs in Sera of EAE Mice

We then quantitated the levels of the above nine miRNAs, which were identified based on microarray miRNA expression analysis, in serum samples of normal (naïve) controls and EAE mice. Of these nine miRNAs, miR-99b, miR-125a, and miR-146b were found to be significantly higher in the EAE mice than the normal mice (Figure 3). This pattern is in agreement with the results obtained by microarray analysis. Similarly, four other miRNAs (miR-23b, miR-31, miR-193b, and let-7e) showed a directional match (increase in EAE mice) with the results of microarray analysis, but the difference was not statistically significant.

### 2.4. The mRNA Targets of Select miRNAs Identified in EAE

Further analysis and literature search revealed several mRNA targets of these nine miRNAs (Table 1). These targets genes were selected from a larger set of mRNAs based on the involvement of the encoded proteins in various functional pathways in the immune-mediated pathological events in EAE/MS. Table 1 shows representative mRNA targets of specific miRNAs. As discussed below (Section 2.6), we examined in detail two of these targets and validated their association with miR-99b and miR-125a.

**Figure 4:**
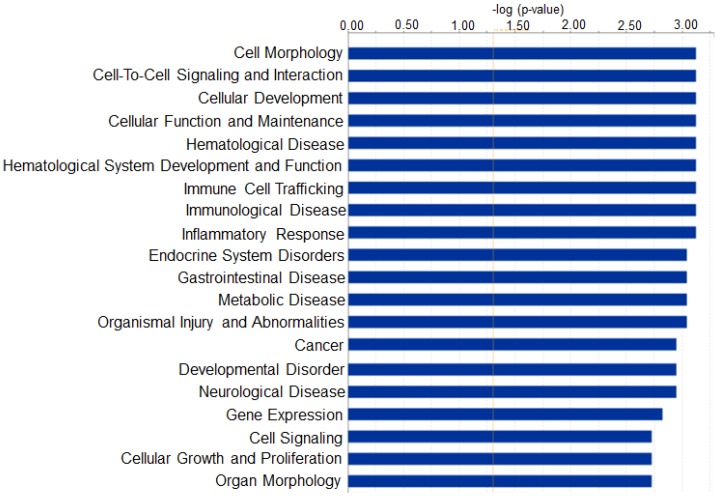
Bar graph indicating the number of miRNAs that are predicted to target genes in each category listed on the *x*-axis. Here, Ingenuity Pathway Analysis (IPA) provides information about which pathways can potentially be influenced by the top miRNAs.

To examine the potential biological functions of the select miRNAs, the data was subjected to Ingenuity Pathway Analysis (IPA) Core analysis. The selected genes were then further grouped into functional categories and plotted as a bar graph (Figure 4). This analysis revealed several different functional categories including cellular development, immunological disease, inflammatory response, neurological disease, and others. As in other diseases, certain miRNAs can participate in different pathophysiological events in more than one organ, and therefore, it is not uncommon that IPA analysis reveals additional diseases besides EAE/MS that might be associated with certain miRNAs.

### 2.5. The mRNA Targets of Select 3 Top miRNAs

**Figure 5:**
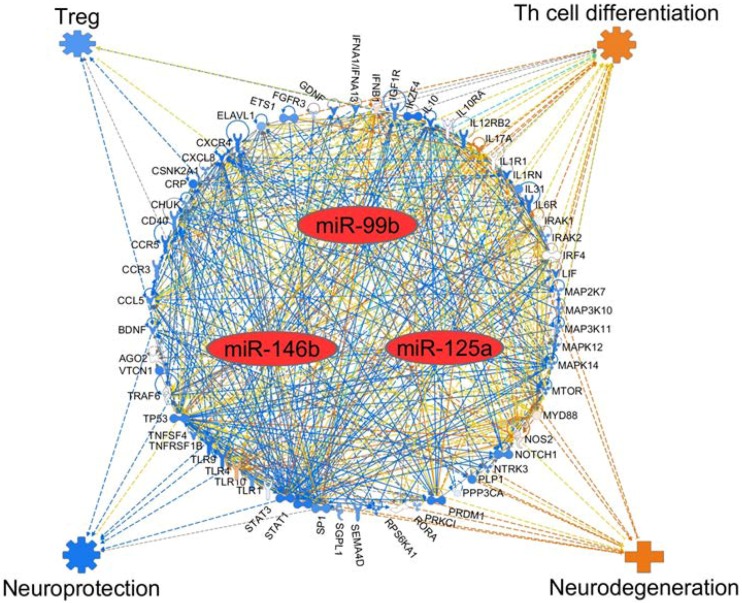
Network analysis of select miRNAs and their target mRNAs, as well as their impact on the disease process in multiple sclerosis. IPA-based schematic representation of various mRNA targets affected by the three major miRNAs altered during EAE development, namely miR-99b, miR-125a, and miR-146b, leading to differential effect on pathogenic versus protective T cell subset as well as neuroprotection versus neurodegeneration. The known interactions between the genes are represented by lines showing activation (arrow) or inhibition (blunt end). Further, solid line indicates direct interaction, whereas dashed line indicates indirect interaction. Colored lines indicate the following: orange line for activation, blue line for inhibition, yellow line for uncertain state of the downstream molecule, and gray line for effect not predicted. For the target genes, orange indicates predicted activation, whereas blue indicates predicted inhibition.

**Figure 6:**
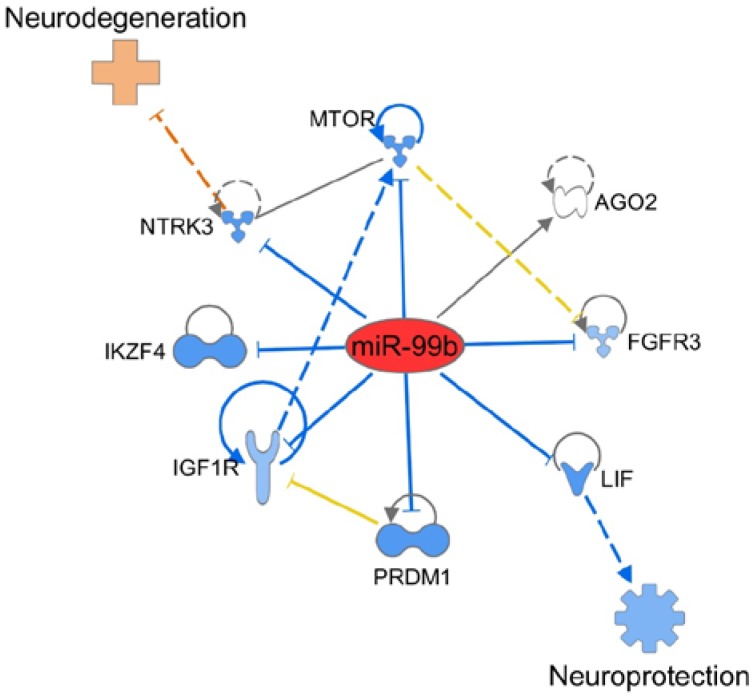
IPA-based schematic representation of the mediators and pathways affected by miR-99b, leading to neuroprotection versus neurodegeneration depending on the direction of change. The description of lines, arrows, color, etc. is same as in the legend to Figure 5.

Next, we used IPA software to determine the potential gene (mRNA) targets of three miRNAs (miR-99b, miR-125a, and miR-146b) that showed significant alteration in EAE mice. The results are shown in Figure 5. Many genes potentially relevant for MS were evident in that analysis. That analysis further revealed the likely impact of those genes on two critical aspects of EAE pathogenesis, namely the differentiation of pathogenic (e.g., T helper 17 (Th17)) and protective (e.g., T regulatory (Treg)) cells, as well as protection versus damage to the neuronal tissue. Interestingly, miR-99b, which our study has revealed to be a novel miRNA for EAE about which there is little information in EAE literature, modulates certain key genes involved in the immune pathology of the neuronal tissue (Figure 6).

### 2.6. BDNF and/or LIF Represent Key Targets of miR-125a and 99b

**Figure 7:**
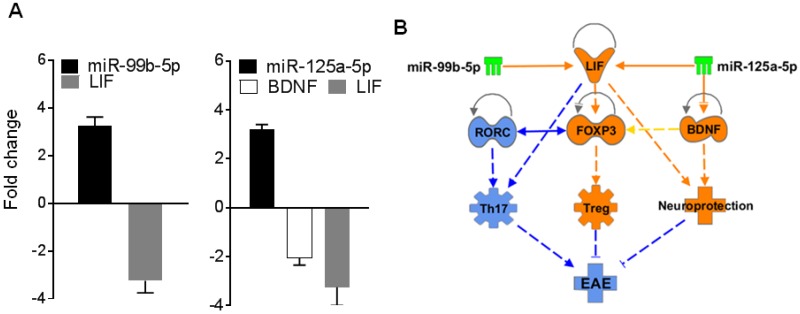
(**A**) Total RNA isolated from splenocytes of EAE mice at peak phase of the disease was tested by qRT-PCR for the indicated miRNAs and their potential gene targets. The results are expressed as ‘Fold change’ over a housekeeping gene; (**B**) IPA analysis of BDNF, LIF, and other mediators and pathways pertaining to EAE that are modulated by miR-99b and miR-125a.

**Figure 8:**
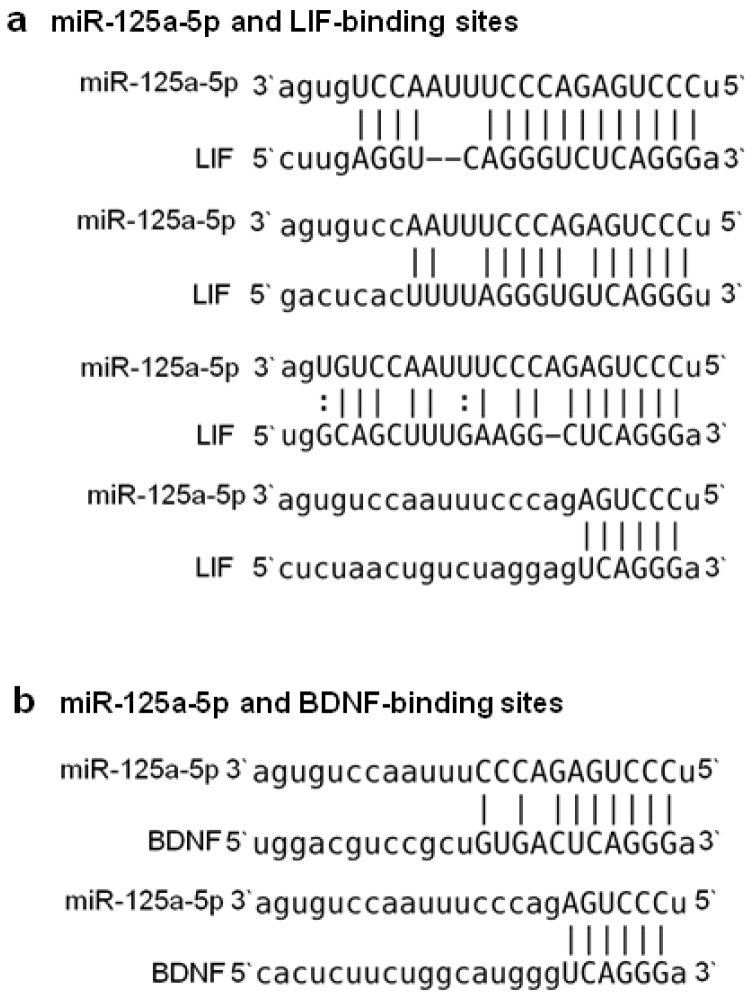
miR-125a has binding sites for 3’UTR of mRNA for BDNF and LIF.

From data shown in Table 1 and additional bioinformatics analysis, we selected two targets for further testing. These targets are brain-derived neurotrophic factor (BDNF) and leukemia inhibitory factor (LIF), both of which are known to be associated with neuroprotection in EAE [36,37,38,39]. We reasoned that because of the observed increase in the above-mentioned two miRNAs in EAE mice, there should be a corresponding decrease in the two target genes (*BDNF* and *LIF*) in the same splenocytes. To test this proposition, we tested the total RNA derived from splenocytes of EAE mice harvested at peak phase of the disease by qRT-PCR for levels of miR-99b, miR-125a, *BDNF*, and *LIF* following the methods described below. The results are shown in Figure 7A. Interestingly, as predicted, there was an inverse correlation between miR-125a and *BDNF* as well as *LIF*, and between miR-99b and *LIF* (Figure 7A). These results demonstrate at least one mechanism by which these two miRNAs influence EAE development. IPA analysis further pointed to the association between these two miRNAs and the two mRNA targets in the disease process (Figure 7B). In addition, it was evident that miR-125a has binding sites for 3’ untranslated region (3’UTR) each of mRNA of BDNF as well as LIF, supporting our experimental results shown in Figure 8. (However, because of insufficient information for miR-99b to conduct this association, we limited our search to miR-125a at this time.)

## 3. Discussion

MS is a complex disease whose pathogenesis involves a variety of mediators that lead to the inflammation and demyelination of the neurons in the CNS. These mediators and their interactions with each other are controlled by many factors, including miRNAs. As mentioned above, there are three main tests currently in use to diagnose and monitor MS: the oligoclonal bands in the CSF [12]; the white matter/Gad-enhancing lesions on MRI [13,14]; and the John Cunningham (JC) virus antibody titers tested in a subset of MS patients [15,16]. While these tests have been used extensively, they have inherent limitations of specificity and sensitivity. For example, the white matter lesions have only been associated with MS patients in a certain score range [17], and there is a weak correlation between the Gad-enhancing lesions and cognitive decline in relapsing-remitting MS (RRMS) [19]. Moreover, while approximately 50% of MS patients test seropositive for JC antibody titers, less than 1% might develop progressive multifocal leukoencephalopathy (PML), a rare side effect of treatment with drugs such as Tysabri (Natalizumab) [15,18]. Therefore, better biomarkers are required to diagnose MS early to benefit the patients by starting the appropriate treatment early. Efforts are being made to identify and validate new serum/plasma biomarkers for MS [40] and other neurological diseases such as Alzheimer’s disease [41]. In this context, we believe that miRNAs not only play an important role in modulating the disease process in MS, but also can serve as new biomarkers in conjunction with current biomarkers for this disease. EAE is a widely used mouse model to study the pathogenesis of MS [33,34]. The miRNAs selected in our current study (miR-99b, miR-125a, and miR-146b) are predicted to control the target genes important for EAE pathogenesis, and therefore can serve as biomarkers of EAE development.

In this study, we examined the miRNA profile of splenocytes of EAE versus control mice. We used miRNA-microarray technology in conjunction with a bioinformatics-based approach to determine the miRNA profiles in the splenocytes of these mice. After extensive analysis using IPA, Targetscan, and other software, we selected nine miRNAs for further analysis: let-7e, miR-23b, miR-31, miR-99b, miR-125a, miR-146b, miR-155, miR-193b, and miR-221. Two of the notable miRNAs are miR-99b and miR-125a. Of these, miR-125a has been the subject of a few reports for its immunomodulatory effects in MS/EAE as discussed below [42,43,44,45]. However, to the best of our knowledge, the importance of miR-99b in EAE/MS has not yet been appreciated. These two miRNAs, along with seven others, are increased in splenocytes of mice upon EAE development. Importantly, the significance of some of these miRNAs is further evident from the results of serum testing, which showed that miR-99b, miR-125a, and miR-146b were significantly higher in the sera of EAE mice than normal mice. These miRNAs represent potential circulating biomarkers of disease activity.

The novel finding of our study is the involvement of miR-99b in EAE. Although several different miRNAs have been shown to be altered in mice with EAE and patients with MS, there is barely any information on miR-99b in EAE. A recent report described the role of miR-99b and mammalian target of rapamycin (mTOR) signaling in neurodegeneration in mice after spinal cord injury [46]. Although that study was not conducted in the EAE model, it indirectly supports the association between miR-99b and spinal cord inflammation, which also is a component of EAE. Interestingly, an association between miR-99b and mTOR has also been observed in cancer cells [47]. In regard to MS, miR-99b was among 12 miRNAs that were found to be increased in pediatric MS [43]. Although that study was limited to the detection of altered miRNAs and their correlation with specific mRNAs, it supports our finding of the involvement of miR-99b in EAE pathology. Furthermore, our results show that miR-99b might regulate LIF, which has been shown to have neuroprotective effect in EAE [38,39]. Increase in miR-99b during EAE inhibits LIF, which thereby inversely correlates with disease development. We believe that our study has unraveled a novel role of miR-99b in the immunopathogenesis of EAE as well as its potential as a biomarker of disease. Another miRNA of interest revealed in our study is miR-125a, which is discussed below.

Our results showed that miRNA-125a is increased in splenocytes of EAE mice. This finding is supported by results of two studies in MS patients that reported an increase in miRNA-125a [42,43]. This miRNA has been shown to inhibit oligodendrocyte maturation, and thereby can contribute to the disease process [43]. However, another study revealed that miRNA-125a is a regulator of brain endothelial tightness as well as infiltration of the barrier by immune cells, and that reduced expression of this miRNA can lead to enhanced pathology in MS [42]. Two other studies have examined the effect of miRNA-125a on Treg and/or T effector cells. In a study in EAE, a synthetic miR-125a analog inhibited various effector T cell factors (e.g., signal transducer and activator of transcription 3 (STAT3), interferon gamma (IFNγ), and IL-13) and showed the potential of this miRNA to reprogram the immune system and return it to homeostatic conditions [45]. In another study, miR-125a was shown to be an important stabilizing molecule for the Treg phenotype [48]. Under steady-state conditions, miR-125a expression was low, but in an inflammatory environment, the GATA-binding protein 3 (GATA3) increased the miR-125a levels. This then led to a decrease in the expression of IL-6 receptor and STAT3, and thereby inhibited the conversion of Treg into inflammatory-type T cells [48]. These latter two studies suggest that an increase in miRNA-125a correlates with higher immune suppression. It is likely that the observed differences might in part be owing to different cell types studied, example Treg versus splenocytes. In addition, the timing of miRNA testing can also affect results because of differences in the kinetics of expression of miRNAs in various tissues. Also, as a single miRNA has the capacity to target several genes, the regulation of one set of genes, example those involved in T cell differentiation, might differ from that of neurotrophic factors, etc. Nevertheless, taken together, these studies highlight the significance of miRNA-125a in MS/EAE. Our results described above further highlight the involvement of miR-125a in EAE. We observed that miR-125a can regulate BDNF and LIF, both of which are associated with neuroprotection. An increase in miR-125a following EAE induction is then expected to inhibit BDNF and LIF, which show an inverse association with disease development.

We observed an increase in miR-155 in EAE. Our results are supported by those of previous studies on this particular miRNA. It has been shown in the EAE model that miR-155 can affect the differentiation of T cells and skew the T cell response towards a proinflammatory phenotype by producing more Th1/Th17 cells [49]. Furthermore, it has been shown that silencing miR-155 in CD4+ T cells resulted in a reduction in morbidity in EAE mice compared to controls [50]. Similarly, protection induced in EAE by high dose IL-17 (a seemingly paradoxical effect) was associated with reduction in miR-155 [51]. Moreover, increased levels of miR-155 in endothelial cells mimicked cytokine-induced increase in the permeability of the blood brain barrier in EAE, which in turn can potentially enhance CNS inflammation [52]. In regard to MS patients, one study reported an increase in miR-155 that correlated with the disease [53], whereas another showed that miR-155 was higher in remission phase than in relapse phase [54]. Furthermore, miR-155 is also a target of some of the current disease-modifying treatment regimen in MS [55].

We have described above three of the nine miRNAs that were significantly altered in EAE. Of the remaining six, three miRNAs (let-7e, miR-23b, and miR-31) have been reported to be involved in EAE [56,57,58]. In one study on EAE, miRNA let-7e was found to be increased in expression mainly in CD4+ T cells and mononuclear cells infiltrating the CNS. Interestingly, silencing of let-7e in vivo resulted in inhibition of Th1 and Th17 cells, but upregulation of the Th2 cell response, leading to attenuation of EAE. The overexpression of let-7e had an opposite effect [56]. In a set of other studies, miR-23b was shown to influence EAE by suppressing IL-17 response by targeting TGF-β-activated kinase 1/MAP3K7 binding protein 2 (TAB2), TAB3 and inhibitor of nuclear factor κ-B kinase subunit α (IKK-α) [59], or via suppressing leukocyte migration by targeting the chemokine (C-C motif) ligand 7 (CCL7) [57]. These results differ from ours mentioned above that state miR-23b is increased in EAE mice. The precise reasons for this difference need further evaluation. Finally, a third study showed increased expression of miR-31 in CD11c+ dendritic cells (DCs) but not LysM+ microglia in mice with EAE [58]. It was concluded from bone marrow chimera experiments that the expression of miR-31 was specific to bone marrow-derived DCs. This miRNA influenced EAE development by primarily regulating the migration of DCs into the CNS. 

The remaining three miRNAs in our study are miR-146b, miR-221, and miR-193b. Of these, miR-146b has been shown to be increased in MS and linked with disease development in one study [53], but associated with remission from MS in another study [54]. Furthermore, miR-146b has also been identified in the transcriptome of human Foxp3+ Treg [60]. Similarly, miR-221 was shown to be increased in pediatric MS [43]. However, to the best of our knowledge, miR-193b has not been reported for EAE or MS. Thus, miR-193b needs further examination and evaluation. In addition to above-mentioned miRNAs, other investigators have reported the involvement of miR-223 [61] and miR-466i [62] in EAE pathogenesis. Taken together, the results of these studies highlight the significance of miRNAs in the disease process in EAE, which in turn might provide insights into human MS.

## 4. Materials and Methods

### 4.1. Induction of Experimental Autoimmune Encephalomyelitis (EAE) in Mice

Animal handling, animal care, and experimental work involving them were performed according to the Institutional Animal Care and Use Committee (IACUC) guidelines of the, University of Maryland School of Medicine, Baltimore, MD, USA via protocol # 0616004 approved in June 2016. EAE was induced in C57BL/6 mice (JAX, Bar Harbor, ME), 6–8 week old male, following the method described elsewhere [63,64]. Briefly, mice were immunized subcutaneously with MOG_35–55_ peptide (AnaSpec Inc., Fremont, CA, USA) (200 µg per mouse) in complete Freund’s adjuvant (CFA) followed by concomitant intraperitoneal (i.p.) administration of Pertussis toxin (EMD Millipore Corp., Billerica, MA, USA) on day 0 and day 2. Thereafter, these mice were observed regularly for the development of disease. The severity of EAE was graded on a scale of 0 to 5 as described in detail elsewhere (Figure S1) [64]: grade 1 = partial or total flaccid paralysis of tail; 2 = hind limb weakness/disrupted righting reflex; 3 = flaccid paralysis in one hind limb; 4 = flaccid paralysis in both hind limbs; and 5 = moribund/dead. The severity of disease was further confirmed by histological examination of CNS, particularly spinal cord after staining of tissue sections by hematoxylin and eosin (H&E) as well as Luxol fast blue staining (data not shown.)

### 4.2. The Testing of Splenocytes (SPCs) of EAE and Control Mice

Spleens of EAE mice were harvested when the clinical score was between 3 to 4, which on average was about day 17–18 after MOG_35–55_ injection. A single cell suspension of splenocytes was prepared and then it was subjected to the lysis of red blood cells using ammonium-chloride-potassium (ACK) lysis buffer (Quality Biologics, Gaithersburg, MD, USA), followed by three washings with Hanks’ balanced salt solution (HBSS) (Gibco, Gaithersburg, MD, USA). These splenocytes were then plated at 6 × 10^6^ cells/well in a 6-well culture plate in Dulbecco’s Modified Eagle’s medium (DMEM) (Sigma Aldrich, St. Louis, MO, USA) containing 10 % fetal bovine serum (FBS) supplemented with 2 mM L-glutamine, 100 units/mL penicillin G sodium, and 100 μg/mL of streptomycin sulfate in an atmosphere of 95% air and 5% CO_2_. The cells were then restimulated for 24 h with MOG_35–55_ Aliquots of cells cultured in medium only served as ‘Medium control’.

### 4.3. Preparation of Total RNA and Microarray Hybridization for miRNA Expression Analysis

The total RNA was isolated from splenocytes (SPCs) using RNeasy Mini kit (Qiagen, Germantown, MD, USA) following the manufacturer’s protocol. The concentration of the total RNA was determined with a NanoDrop ND-1000 spectrophotometer (Thermofisher, Waltham, MA, USA) at 230, 260, and 280 nm, and the RNA quality was assessed by automated capillary gel electrophoresis on an Agilent bioanalyzer 2100. The total RNA isolated from the SPCs of 3 biological replicates for the test conditions, namely Medium control and MOG_35–55_ restimulation, were used for miRNA expression using GeneChip^TM^ miRNA 4.0 Array (Affymetrix, Santa Clara, CA, USA). Hybridization and data acquisition were performed according to the manufacturer’s instructions.

### 4.4. Micro-RNA Expression Analysis

The raw data for miRNA microarray was preprocessed using the Robust Multi-array Average (RMA) algorithm to perform the background correction. The preprocessed data was then analyzed using Expression Console (Affymetrix, Santa Clara, CA, USA) and other software. The miRNAs, whose expression was significantly altered with a *p*-value of <0.05 in a pairwise comparison of the two conditions mentioned above (Medium control and MOG_35–55_ restimulation), were further selected using the cutoff of *p*-value below 0.05 and fold change >2 or <−2. Principal component analysis (PCA) was conducted on the significant miRNAs identified from the above two conditions. PCA is a statistical tool to assess data quality and define relationships between different sets of data points. The data points belonging to different control and treatment groups are scattered in a 3-dimentional space indicated by 3 axes or principal components (PC), represented as PC1, PC2, and PC3. The percentage value associated with each PC represents the variance captured by each of the 3 components. Data points of one group, which are shown in one particular color, if clustered together away from other data points belonging to another group, that implies that the gene expression profile of different groups are different from each other. On the other hand, if the data points overlap, partly or fully, then that indicates some overlap in gene expression profiles. The results obtained from above analysis were validated by qRT-PCR and serum testing, and therefore, the main conclusions drawn from those results are reliable. The Ingenuity Pathway Analysis (IPA) was conducted on the significant miRNAs from the group comparisons, and then multiple miRNAs and gene interaction networks were captured as described below. In microarray analysis based on hybridization of different probes binding to a given miRNA sequence, the term “miRNA elements” refers to intensity signals obtained using homologous probes of a given miRNA that belong to different species such as mouse, rat, and human. However, the term “miRNA” is used instead of “miRNA elements” when intensity signals of a single miRNA are obtained using probes of a single species such as mouse.

### 4.5. Validation of Mature miRNA by Quantitative Real-Time PCR (qRT-PCR)

Total RNA was used for quantification of mature microRNAs (miRNAs) using TaqMan MicroRNA Assays kit (Life technologies, Carlsbad, CA, USA) according to the manufacturer’s protocol. Using TaqMan MicroRNA Reverse Transcription Kit (Applied Biosystems, Beverly, MA, USA) containing MultiScribeTM reverse transcriptase and dNTPs, single-stranded cDNA specific for miRNA to be tested was synthesized from 10 ng of the total RNA of each sample. Employing sequence-specific primers from the TaqMan Assay Kit and TaqMan Universal PCR Master Mix in an ABI PRISM 7900HT real-time PCR system (Applied Biosystems, Foster City, CA, USA), the cDNA prepared above was amplified. Thereafter, using U6, a constitutively expressed endogenous control miRNA, the specific miRNA levels were normalized and the relative gene (mRNA) expression levels were assessed. The results were expressed as fold change compared to Medium control.

### 4.6. Testing miRNA Levels in Sera of Mice

The levels of miRNAs in sera of normal (naïve controls) and diseased (EAE) mice were tested using Multiplex miRNA assays with FirePlex Particle Technology (Abcam, Cambridge, MA, USA). Briefly, blood samples were collected from mice on days 18–20 after MOG_35–55_ injection for the purpose of induction of EAE. Thereafter, serum was prepared from blood and 20 μL of each sample was subjected to multiplex miRNA assay [65]. The results obtained by Multiplex assay were analyzed using FirePlex Analysis Workbench software (Ver. 2.0.234, Abcam, Cambridge, MA, USA). The data was presented after normalizing with appropriate normalizing controls.

### 4.7. Ingenuity Pathway Analysis (IPA) for Gene (mRNA) Target Prediction and Network Analysis

Ingenuity Pathway Analysis (IPA) software (Ver. 01–07, Qiagen, Germantown, MD, USA) was used to predict gene (mRNA) targets of specific miRNAs, and to create specific molecular interaction networks from the genes that are predicted/validated targets of the top-listed miRNAs. The effects of specific miRNAs and their target mRNAs on T helper cell/Treg differentiation as well as neuroprotection were analyzed using the molecule activity predictor (MAP) tool in IPA. Finally, the top-listed miRNAs that have the potential to target these selected genes were identified.

### 4.8. Measurement of mRNA Expression Using qRT-PCR

Total RNA was isolated from splenocytes as described above, and then cDNA was prepared from RNA using iScript cDNA synthesis kit. The resulting cDNA was amplified in an ABI PRISM 7900HT cycler by qRT-PCR using SYBR Green PCR Master Mix and appropriate primers. The sequences of the forward and reverse primers for the detection of mRNAs were selected from the Primer Depot (National Institutes of Health (NIH)) and the corresponding primers were obtained from Sigma-Aldrich (St. Louis, MO, USA). The mRNA levels of specific genes were normalized to Glyceraldehyde-3-Phosphate Dehydrogenase (GAPDH) mRNA levels, and the relative gene expression levels were determined. The results were expressed as ‘Fold change’.

## 5. Conclusions

Using the EAE model, we identified nine miRNAs of interest, which represent cellular as well as circulating miRNAs. Of these, miR-99b, miR-125a, and miR-146b can serve as potential disease biomarkers. Validation of these miRNAs in a larger cohort of EAE mice and MS patients is currently being planned. In addition, BDNF and/or LIF were identified as two of the gene targets of miR-99b and miR-125a. Detailed studies to unravel the molecular mechanisms regulated by these miRNAs and their target genes are currently in progress.

## Figures and Tables

**Table 1:** The mRNA targets of select miRNAs altered during EAE development in mice.

miRNA	mRNA Targets
Let-7e-5p	AGO2, BCL2L1, BSG, CCL3, CCR7, CD200, CD28, CD80, CD86, CHUK, CRP, DUSP1, EPHA4, FASLG, IL12RB2, IL13, IL6, ITGB8, LYN, MAPK11, PRDM1, RORC, TLR4, TNFRSF1B, TNFSF10
miR-155-5p	CTLA4, CD274, CD47, HIF1A, RELA, SMAD4, ETS1, NDFIP1, IL21, ITK, S1PR1, BDNF
miR-146b-5p	AGO2, CCR3, CCR5, CD40, CHUCK, CRP, CXCL8, CXCR4, GDNF, IFNA1/IFNA13, IFNB1, IKZF4, IL10, IL17A, IL1R1, IL2RB2, IRAK1, IRAK2, IRAK2, MAPK14, NOS2, PLP1, SP1, STAT1, STAT3, TLR1, TLR10, TLR4, TLR9, TRAF6
miR-125a-5p	AGO2, BDNF, CCR5, CSNK2A1, ELAVL1, ETS1, IKZFA, IL10RA, IL1RN, IL31, IL6R, IRAK1, IRF4, LIF, MAP2K7, MAP3K10, MAP3K11, MAPK12, MAPK14, MYD88, NOS2, PRDM1, RPS6KA1, SEMA4D, SGPL1, STAT3, TLR4, TNFRSF1B, TNFSF4, TP53, VTCN1
miR-99b-5p	AGO2, FGFR3, MTOR, NTRK3, IGF1R, LIF, PRDM1, IKZF4
miR-221-5p	IFNAR1, HTT, IL6ST, BDNF, IRF4
miR-23b-5p	CCL7, FOXO4, IKKA, SMAD3, TAB2, TBA3
miR-193b-3p	KIT, SOX5, BCL2L2, ETS1, SOCS3, ALOX5, KIT
miR-31-3p	PRDM1, NFATC2, HAVCR2, STAT3, FOXO1, FOXP3

## Supplementary Materials

Supplementary materials can be found at http://www.mdpi.com/1422-0067/19/12/3990/s1.
