# Supplementary material for: The miRNA Expression Profile of Experimental Autoimmune Encephalomyelitis Reveals Novel Potential Disease Biomarkers
Source: Int J Mol Sci. 2018 Dec 11;19(12):3990. doi: 10.3390/ijms19123990 (PMC6321564; doi:10.3390/ijms19123990)
Supplement: Supplementary file 1 [file ijms-19-03990-s001.pdf]

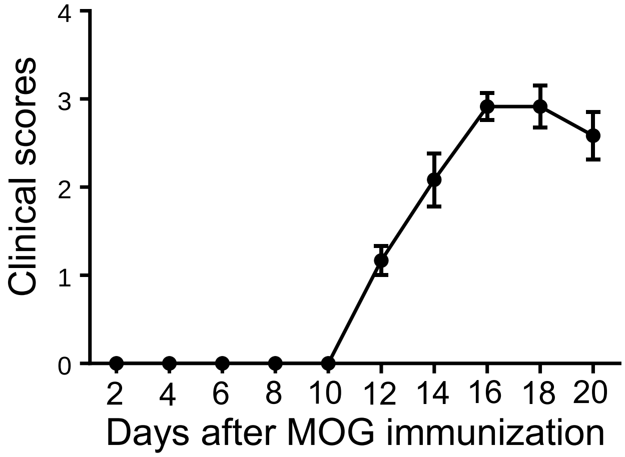

Supplementary Figure 1. A representative profile of EAE in C57BL/6 mice. EAE was induced in C57BL/6 mice by subcutaneous immunization with MOG_{35-55} peptide (200 ug per mouse) in complete Freund's adjuvant (CFA) (5mg/ml) followed by concomitant intraperitoneal (i.p.) administration of Pertussis toxin on day 0 and day 2. Thereafter, these mice were observed regularly for the development of disease. The severity of EAE was graded on a scale of 0 to 5 as follows: grade 1 = partial or total flaccid paralysis of tail; 2 = hind limb weakness/disrupted righting reflex; 3 = flaccid paralysis in one hind limb; 4 = flaccid paralysis in both hind limbs; and 5 = moribund/dead. EAE scores (mean ± SEM) are shown.
